# A novel lightweight deep learning model for early prediction of cardiovascular disease

**DOI:** 10.3389/frai.2026.1828950

**Published:** 2026-05-20

**Authors:** Nasir Gul, Aurangzeb Khan, Mazliham Mohd Su'ud, Muhammad Mansoor Alam, Danyal Skandri

**Affiliations:** 1 University of Science and Technology, Bannu, Bannu, Pakistan; 2 Faculty of Computing and Informatics, Multimedia University, Cyberjaya, Malaysia; 3 Riphah International University Information Services, Islamabad, Pakistan

**Keywords:** ANN, CVD, deep learning, machine learning, oversampling, random forest, risk prediction

## Abstract

Cardiovascular Disease (CVD) is still the main cause of death globally. Hence, to have timely clinical intervention, there is a need for the prediction of early risk models which are accurate and dependable. Here is a lightweight and strong artificial intelligence-based system for the early prediction of CVD using clinical data. We have created an end-to-end method comprising data preprocessing, stratified train and test splitting, and class imbalance treatment by SMOTE exclusively on the training set to ensure that there is no data leakage. We evaluate a series of machine learning (ML) and deep learning (DL) models, including Logistic Regression (LR), Decision Tree (DT), K-Nearest Neighbors (KNN), Random Forest (RF), Support Vector Machine (SVM), and various Artificial Neural Network (ANN) architectures, systematically. In order to check the robustness and generalizability of the models proposed, we conduct a k-fold cross-validation procedure and the performance measures are reported using evaluation matrices such as accuracy, precision, recall, F1-score, and ROC curve along with statistical metrics (mean and standard deviation). The results from the experiments show that the fine-tuned four-layer ANN yields the best prediction performance. Nevertheless, and in contrast to conventional studies, evaluation of the models is strongly based on robust validation and statistical reliability and not only on accuracy.

## Introduction

Heart diseases, or CVDs, are the third most prevalent diseases in the globe and a major cause of death. According to a survey presented by the World Health Organization (WHO) in June 2012, 17.5 million total worldwide fatalities occurred due to heart attacks and brain strokes (BS). In addition, more than 75% of fatalities from CVDs happen mostly in developing and power nations. Furthermore, 80% of the causalities that happen because of CVD are due to heart attack and BS. It is seen as the second epidemic in many countries, replacing infectious diseases as the major cause of deaths globally in males and females above the age of sixty. CVDs include coronary heart ailment, cerebrovascular ailment, rheumatic heart ailment, and other conditions affecting the heart. Smoking, a bad diet, absence of physical activities, and too much alcohol drinking usually lead to them. In this study, we will be using ML and DL approaches to forecast cardiovascular disease rates on a global scale (Reynolds et al., 2017).

Heart failing is the situation where the heart loses its ability to pump enough oxygen-rich blood to meet the needs of the body. This leads to a storage of fluid, breathing problems, and swelling in the lower legs (peripheral edema). Central edema happens when the brain gets too much fluid and can result in mental confusion or drowsiness. Heart failure affects men and women equally and can happen at any age. Symptoms vary from person to person; however, they can include shortness of breath, chest pain, unusual weight gain or loss, fatigue, weakness or numbness in one arm or leg; dizziness or fainting episodes; and needing to urinate more often than normal. It is caused by high cholesterol, high blood pressure, and other chronic diseases. Therefore, a large number of Americans have reduced their risk factors for heart disease by eating healthier foods and maintaining physical activity. As most people do not understand the severity of heart disease, they often miss or forget to check their hearts. Similarly, people who feel symptoms of cardiac issues or discomfort do not treat them which can lead to an early stage of heart failure associated with chronic symptoms like tiredness and fatigue. Treating cardiac issues early means more chances of survival, less medication, and no downtime because it has been treated while it’s still early in its course (Nagavelli et al., 2022).

CVDs, including heart attack, stroke, cardiomyopathy, and heart failure (HF), are among the common causes of fatalities in most developed countries (Troesch et al., 2015). These conditions can occur at any age but are more prevalent in older people. One-third of all cardiac diseases are caused by diabetes, while obesity contributes to almost half of CVD cases worldwide. According to WHO statistics (2017), about 300 million deaths from CVDs occurred worldwide, which makes it one of the major contributors to global disability (Nashif et al., 2018).

Identifying risk factors such as diet, blood pressure, and blood sugar is crucial for positive health outcomes. Dietary patterns strongly influence metabolic syndrome and obesity-related disorders, potentially leading to chronic conditions like type 2 diabetes. Healthy eating is linked to improved cardiovascular health, while poor diets increase mortality rates. Despite this, few reviews assess dietary patterns’ long-term impact on cardiovascular outcomes (Mantoani et al., 2017). Food intake patterns, particularly nutrient content, play a key role in CVD onset and severity. Researchers emphasize the importance of essential nutrients like vitamin B12, zinc, and niacin, highlighting the need for balanced diets to mitigate CVD risks (Heron, 2013; Princy et al., 2020; Hannan et al., 2010; Shouman et al., 2012).

An early-stage diagnosis of heart diseases can help reduce the rate of mortality (Kim et al., 2022). The recognition of CVDs initial signs is mostly based on human assessment, leading to various issues such as inaccurate identification, subjectivity, and missing small differences, as well as increasing diagnostic time (Taye, 2023). AI-based evaluation and diagnosis is an alternative to conventional methods that is more accurate, free of subjectivity, has the ability to detect minor differences, and produces fast outcomes useful for patient care and treatment. The introduction of AI-based techniques improved the precision and speed of illness detection, and scholars are still working to incorporate new processes into the current frameworks, such as ML and DL approaches, to revolutionize the area of healthcare and lessen the load on medical experts. The majority of earlier studies used ML techniques to forecast early signs of illnesses via demographic data and regarded them as resource-effective and also precise due to the structure of the data’s intricacy, wherein ML methods perform poorly in comparison with DL methods. In the past 10 years, DL methods have been regarded as more effective and generalizable in predicting early signs of illness via data on demographics (Patel et al., 2016). The modern approaches to CVD diagnosis rely on smart tools, and experts have been applying new model structures to improve illness identification ratios and generalization (Jagtap et al., 2019). Techniques based on DL have greatly enhanced robustness and accuracy, leading to better patient care and treatment (Bahrami and Shirvani, 2015). The improvement in the healthcare field based on smart AI tools has proven to be a positive step towards precision diagnosis and care.

The primary goal of this study is to develop a highly accurate model with reduced training parameters that detects early indicators of CVD, giving medical practitioners valuable tools for early identification of CVDs. ML and DL models are utilized to detect abnormalities at an early stage related to CVDs. In this research, various ML approaches such as LR, DT, KNNs, RF, SVM, and various structures of ANN were trained and tested with the highest results from 5-layer ANN with an accuracy of 99%. A public dataset collected from Kaggle followed by various preprocessing techniques was utilized for training and testing the models. By overcoming category inequality via oversampling, the algorithms achieved notable results enhancements, showing the potential of our proposed models in correctly predicting CVD risk early signs. Such techniques can enable more precise and timely early-stage diagnosis and, ultimately, lead to better patient outcomes.

## Literature review

In this section, we present the latest earlier work on the application of ML and DL for the identification and prevention of CVDs. We discussed the various structures of ML and DL approaches that have been applied to the study of CVD, including their advantages and limitations. Through this review, we aimed to provide a comprehensive understanding of the current state of the area and highlighted the potential of ML and DL in improving the prevention and management of CVD. The study in Nagavelli et al. (2022) described an ML approach for predicting cardiac disease. In this article, a quick summary of various ML approaches for detecting heart disease is provided. The study used the Nave Bayes (NBs) method with a weighted strategy to forecast early symptoms of CVD. They also employed another method to automatically analyze, localize, and diagnose ischaemic cardiac abnormalities based on the frequency spectrum, spatial range, and data theory. The study in Troesch et al. (2015) used two ML techniques including SVM and XGBoost for CVD’s early symptom prediction. The SVM was enhanced via dual robustness approach for automatically recognizing heart abnormalities.

Nashif et al. (2018) conducted a research study on the classification of CVD by utilizing different ML approaches. The purpose of the study was to reduce mortality rate via early prediction of heart illnesses and ongoing health monitoring by medical experts. To detect imminent heart disease utilizing ML methods, an initial design for a cloud-based CVD identification system had been put forth in this study. An effective ML approach that was built via a specialized analysis among various ML algorithms utilizing WEKA a Java Based free data interpretation Platform, for the accurate identification of heart ailment. The performance of the suggested CVD classification approach was examined utilizing two famous public datasets and during training, k-fold cross-validation technique was employed. The SVM approach obtained a mean average precision (mAP) of 91.50% and sensitivity and specificity of 97.53 and 94.96%, respectively. Princy et al. (2020) conducted a study utilizing ML algorithms to predict cardiovascular illness. In this research, many ML algorithms were used to identify CVDs. Based on the results, the DT classification strategy outperformed all competitors, such as KNN, RF, LR, NB, and SVM-based methods, in terms of CVD identification. The DT achieved the greatest results, achieving a 73% precision on the test dataset.

Hannan et al. (2010) conducted research to predict the CVD by physical performance measures. The objectives, although chronic obstructive respiratory disease (CORD) frequently coexists with CVDs, it is not yet understood how to more accurately estimate CVD risk in those with CORD. Shouman et al. (2012) used radial basis function (RBF) to interpret healthcare records for heart disorders. The study contained 300 instances of heart patients gathered Sahara Health Centre in Aurangabad. The RBM was used to assess the data of the patient from health records and provide the appropriate medical treatment for CVDs. The results of the study revealed that the RBF was able to correctly predicting the prescribed treatment for heart disease and outperformed the other techniques examined in the proposed work. The study in Kim et al. (2022) used KNN to automatically diagnose heart disorders. The outcomes showed the scope of both KNN and ensemble learning methods. Although, it was noticed that KNNs could only be used to closely specified classes. It shows that, although KNNs may be useful for detecting certain kinds of heart condition, it may not be applicable to more complex instances. Despite this disadvantage, the authors believe that the KNN algorithm, can be an effective tool in the detection of cardiac disorders, particularly when dataset is limited or much specialized. The authors also indicate that KNN can be combined with other approaches to improve accuracy.

Academic scholars have developed a variety of data interpretation approaches to assist medical professionals in classifying cardiac problems. As a result, using novel data identification algorithms may require fewer physical tests. A quick and efficient detection tool is required to reduce the number of casualties resulting from cardiovascular diseases and brain stroke (BS). The study in Taylor et al. (2019) used multiple ML variations, such as the DT algorithm, to find the most effective outcome in heart disease detection using the free and powerful platform WEKA. The methods used were J48, LR, and RF. The outcomes revealed that the J48 approach, a form of DT, has the best accuracy, recall, and precision over the other three approaches. The study in Liu et al. (2019) conducted a ML technique to detect initial symptoms of CVDs. According to this work, inadequate analytical methods render it laborious to identify non-visible relationships and trends in CVDs feature sets. An automatic technique to establishing clinical diagnostics could increase the efficiency of cost-cutting measures. Based on data via Kaggle and a medical study provided through the Cleveland Organization, particularly in the field of cardiac abnormalities, this web-based application with a machine learning (ML) algorithm at the backend attempts to predict CVD symptoms.

Gudadhe et al. (2010) evaluated various machine learning classifiers to a bagging-based system for detecting heart abnormalities. The study found that the bagging strategy outperformed other methods in terms of efficiency ratio and precision limit. This approach works by selecting numerous examples from a training set and learning them with various kinds of characteristics and data values. The bagging method then examines the results, selecting the framework with the higher recognition rate as the best. This provides higher precision than other basic ML techniques and ensures better performance. The proposed bagging technique can also be applied to other classification problems, including forecasting recognizing feelings or stock market fluctuations. Thus, this study demonstrates the bagging algorithm’s potential and usefulness in addressing a range of classification tasks.

Sharan and Sathees Kumar (2016) investigated the efficiency of DT, KNN, SVM, and RF algorithms in the cardiac anomaly diagnosis system. The DT classifier outperformed the other approaches, achieving an accuracy rate for prediction of 98.83%. Medhekar et al. (2013) identified the medical consequences of a brain stroke using unbalanced and partial physiologic data. Before categorization, empty attributes were handled using random forest regression. After that, through ANN model, automated hyper parameter optimization (AutoHPO) method was used on an imbalanced set of training data to identify brain stroke. The data, which included 43,400 patient records and 783 instances of BS, was employed for the studies. When comparing the prediction approach to other preceding methods, the number of false positives ratio was reduced by a mean of 51.50%, or nearly 19.1%. Regarding the outcomes stated, the resulting false positives, precision, and accuracy are 33.1, 71.6, and 67.4%, respectively. Ajam (2015) developed a decision assistance framework for CVD classification using an ML technique based on augmented SVM and ANN. A multilayered perceptron artificial neural network (MLPANN) of five layers, comprising layers for input and output, was designed and trained using the back propagation approach. The results showed that MLPNN performed well when compared to enhanced SVM, demonstrating that it may be effectively used for training and forecasting heart disease.

Muhammad et al. (2020) elaborated the use of data assessment techniques in knowledge discovery in databases to accurately predict heart disease with fewer attributes, using the WEKA open source tool. J48, a freely available Java implementation of the C4.5, was used for its information gain capabilities. A naive Bayes classifier was used to create models with predictive capabilities, while CART was employed to quickly show vital data inter-relations. Results showed that J48 was the fastest algorithm to execute in “8/100 s” while CART showed the highest prediction score of 92.2%. Jeena and Kumar (2016) presented a novel technique based on ML classifiers to predict heart disease. The dataset utilized was the Cleveland Medical Institution groundwork CVD dataset, which contains three hundred and three records with 14 attributes. This approach performs in two phases: training and validation. The training step is based on supervised classification, while in the testing phase the identification of new and unseen records of CVD are provided for assessment. The NB algorithm, based on the Bayesian criteria, was used as the classifier. The outcomes show that the precision of this system can be enhanced by updating the training strategy iteratively.

Rahman et al. (2022) utilized ANNs for effective prediction of heart abnormality. The architecture of ANN is composed of numerous neurons connected to each other and are used to process the input values and extract valuable information from the input data that can be used in decision making process. The structure of an ANN depends upon the number of layers and neurons per layer. ANNs are widely employed in automatic disease diagnosis and enhancement of overall healthcare sector because of their strong prediction power as identifiers, sensitivity for fault, and learning capabilities. The dataset utilized in this study is collected from the Cleveland open source dataset platform and contains 14 attributes and 303 instances. ANNs are built and trained utilizing the back propagation knowledge gaining approach on the training data, with the training and target features being split into a 60 and 20% training and validation set, respectively. The activation mapping utilized is a tangent sigmoid for middle layers and a linear transformation for the final layer. After training, the mean square error (MSE) is computed to be 0.1072 and the prediction accuracy for CVD is 88%. Our goal is to figure out an ML/DL strategy that can accurately predict cardiac disease while containing fewer parameters and being computationally efficient. Intelligent and autonomous identification is a novel technique for extracting non-visible traits and features from large training data that combines analytical and artificial intelligence (AI) approaches.

In the last few years, prediction of cardiovascular disease (CVD) has mainly featured the creation of lightweight, robust, and interpretable deep learning models. These models are aimed especially at overcoming issues in real-time deployment and clinical trust. As a matter of example, Khan et al. (2023) designed a very computation-friendly deep learning system for structured clinical data. Their experiments showed that a less complex model can still perform very well and is even fit for limited-resource situations like edge devices and remote healthcare.

At the same time, model interpretability has been recognized as an essential feature for clinical rollout. Sharma et al. (2022) brought in SHAP-based explainability to their machine learning models, which allowed them not only to interpret prediction globally but also instantaneously at the individual level. Their work proves that explainable models significantly increase clinician’s trust and help them make decisions more effectively in life-threatening situations.

Besides that, enhancing model robustness and generalization capability is a top priority in research. Li et al. (2024) combined the ensemble deep learning method with thorough cross-validation. Their results showed that their model became more stable in predicting and overfitting less when the data distribution varied. It is even more crucial in medical settings where the data samples are not only few but also different from one another.

On top of that, Rahman et al. (2023) took a look at SMOTE-based hybrid learning and stressed out that if the oversampling method is done before data splitting, then data leakage will happen and results will get artificially better. Their work serves as a reminder to carefully follow the protocol when conducting the experiment to get unbiased results.

Besides that, Zhang et al. (2025) pointed out the importance of the use of external validation with the help of several datasets as they found that models tested on only one dataset usually do not work well when put into real-world hospital environment.

Besides these, a number of glaring issues are still prevalent. Many follow-up research has been only directed toward achieving higher accuracy at the cost of validation protocols, or naturalness and reliability without keeping efficiency at the core. Furthermore, a lot of the pieces become victim of data leakage due to faulty execution of resampling methods, are lacking in external validation, and fail to have results fully backed by statistical evidence (i.e., variance, confidence intervals).

As a resolution, this paper work proposes a lightweight artificial neural network (ANN) based framework for the early detection of CVD, focusing on:

- Zero-leakage data preprocessing through the exclusive post-training/test splitting application of SMOTE
- Robust validation through the combination of cross-validation techniques
- Explainability incorporation (SHAP) to increase clinical interpretability, and
- Thorough performance evaluation using several metrics and statistical analysis.

By utilizing this mix together, there will be a trade-off between accuracy, efficiency, interpretability, and generalizability. Hence, the new methodology should be the one that can be easily deployed in real-world clinical scenarios as opposed to the existing ones.

## Proposed method

**Figure 1:**
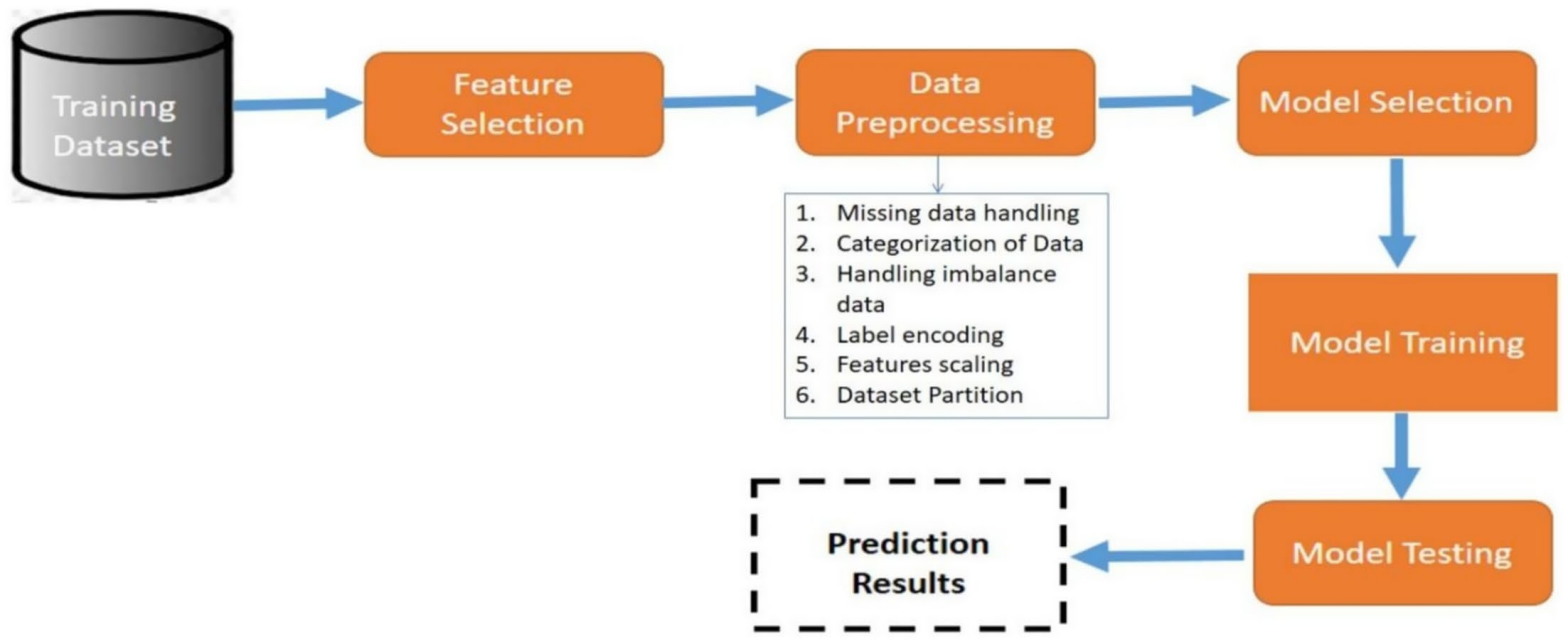
General flow of the study.

The aim of this current work is to build and train an efficient and generalized approach for prediction of CVDs. The study is based on various models, including ML and DL trained and tested carefully to identify the early symptoms of CVDs. The general flow of the proposed work is depicted in Figure 1. The class labels were split into training (80%) and testing (20%) sets through stratified sampling before SMOTE was done. Otherwise, data leakage would have occurred.

In order not to bias the model when performing the testing, SMOTE was done on training data only. Each step of preprocessing was initially performed on the training set, then the same transformations were applied to the test set. The experiments were performed with a set random seed (Dissanayake and Johar, 2021).

### Data collection

The reliability, precision, and performance of an AI model largely depend on the size and diversity of the dataset used for training and testing. To collect a new set of training data from real-scenarios, it needs efforts and expertise. In this approach, we used a freely available training set collected from: https://www.kaggle.com/datasets/jocelyndumlao/cardiovascular-disease-dataset, owned by Jocelyn Dumlao. The dataset contained 14 features with total of 1,000 records with 480 instances with healthy cases while 520 cases contains CVDs signs. Table 1 shows details of features used in this study.

**Table 1:** Shows the details of training data.

S. no	Feature	Type of data
1	Patient id	Numerical
2	Age	Numerical
3	Gender	Binary
4	Resting blood pressure	Numerical
5	Serum cholesterol	Numerical
6	Fasting blood sugar	Binary
7	Chest pain type	Nominal
8	Resting electrocardiogram results	Nominal
9	Maximum heart rate achieved	Numerical
10	Exercise induced angina	Binary
11	Old peak = ST	Numerical
12	Slope of the peak exercise ST segment	Nominal
13	Number of major vessels	Numerical
14	Classification (target)	Binary

### Data preprocessing

To build an AI-based model, several preprocessing techniques are required to clean and prepare the training data. These processes improve data quality, enabling algorithms to concentrate on the text’s relevant patterns and structures. Several strategies were used in this work, including handling of missing data, data balancing using SMOTE, encoding, scaling of features, and dataset portioning. The dataset was analyzed in terms of feature distributions and the presence of missing values. To deal with missing data, we used the [mean/mode imputation] method. Also, to maintain uniformity during the model training phase, all features were normalized via Min-Max scaling.

In order to guarantee a fair comparison, all baseline models were assessed under the same preprocessing, data splitting, and evaluation procedures.

### Missing and incomplete data handling

Conventional imputation techniques such as mean/mode imputation were used in this study for handling missing values. Yet, recent research suggests that patterns of missing data themselves may constitute a source of predictive signal, e.g., Wu et al. (2026) emphasize the notion of informative missingness, where the absence of data is not random but it is related to certain clinical conditions. Taking such mechanisms into account might help improve the performance and understandability of models built on healthcare datasets. Nevertheless, in order to preserve methodological simplicity and computational efficiency, this study uses traditional imputation methods while the modeling of informative missingness will be the subject of future work.

Recent research in EHR data mining indicates that missing data should not always be considered a limitation solely from the technical point of view, as they may provide medically meaningful information. For example, Tan et al. (2023) came up with the idea of informative missingness, which they showed that the lack of lab measurements probably represents underlying clinical decision-making, disease severity, patient pathways instead of random omission. More specifically, their results demonstrate that missingness itself can serve as a predictive indicator and even enhance model performance if it is explicitly incorporated.

This line of thought is in direct agreement with Wu et al. (2026) recent discussions that modeling missingness mechanisms can lead to better predictive modeling of healthcare datasets.

For the current research, to reduce time and solutions of the problem complexity, we resorted to simple and traditional imputation methods (mean/mode) only. Nevertheless, echoing the above-stated studies, we admit that such methods may fail to recognize hidden information from missing data patterns. Thus, the eventual goal of this paper is to work on advanced imputation techniques that might be missingness indicator variables or joint modeling to link with informative missingness and simultaneous improve predictive power and clinical interpretability of the newly developed simple deep learning framework.

Data is an important factor in ML approaches. Missing data is a typical difficulty in interpreting data and can be caused by a variety of factors, including errors in data collection or entry, incomplete surveys, or anomalies in the original gathered data (Kiran, 2024). In this study, the training set was analyzed to correct for all instances of missing data in order to improve model results.

Recent research (Bhandari and Tyagi, 2026) points to the possibility that missing data patterns might be indicative of the outcome. This paper implements conventional imputation methods, however, we intend to take into consideration the informative missingness modeling in our future study to enhance the prediction.

#### Features standardization

This procedure is used for fasting the training process and it guarantees speedy approach towards the minimal loss value. Rescaling in machine learning is a processing of data approach that converts non-normally distributed (SD = 1, average = 0) continuous data points into a distribution that is evenly distributed. In most procedures, the level of scaling of the features influences both the method’s efficiency and convergence time, hence scaling is critical.

#### Feature selection

It is the part of feature engineering and it can be done manually taking help from area experts or built-in scripts can be utilized to show the relationship between various attributes and most interlink attributes can be chosen as features for the said task. Feature selection is the process of picking the most significant qualities, which are the major factors that influence the target anomaly, while eliminating those that play a minor, if any, effect in illness diagnosis. In the suggested study, only the patient_id was removed off the attribute list.

#### Feature encoding

Labelling or feature encoding in ML is a technique for translating text data into a numerical representation, allowing algorithms to process these characteristics. Encoding is essential because it enables algorithms to understand categorical input, which enhances their capacity to identify the valuable information needed for decision making and generate highly accurate results. There are different approaches, each with its own set of advantages and disadvantages, but one must be chosen based on the nature of the task. In this study, Label Encoding was used.

#### Splitting data

Data partition is the technique of splitting training data into two groups, training and validation. In the proposed study, the training data is split in the ratio of 0.80 and 0.20, respectively, for training and validation.

### Balancing training data

**Figure 2:**
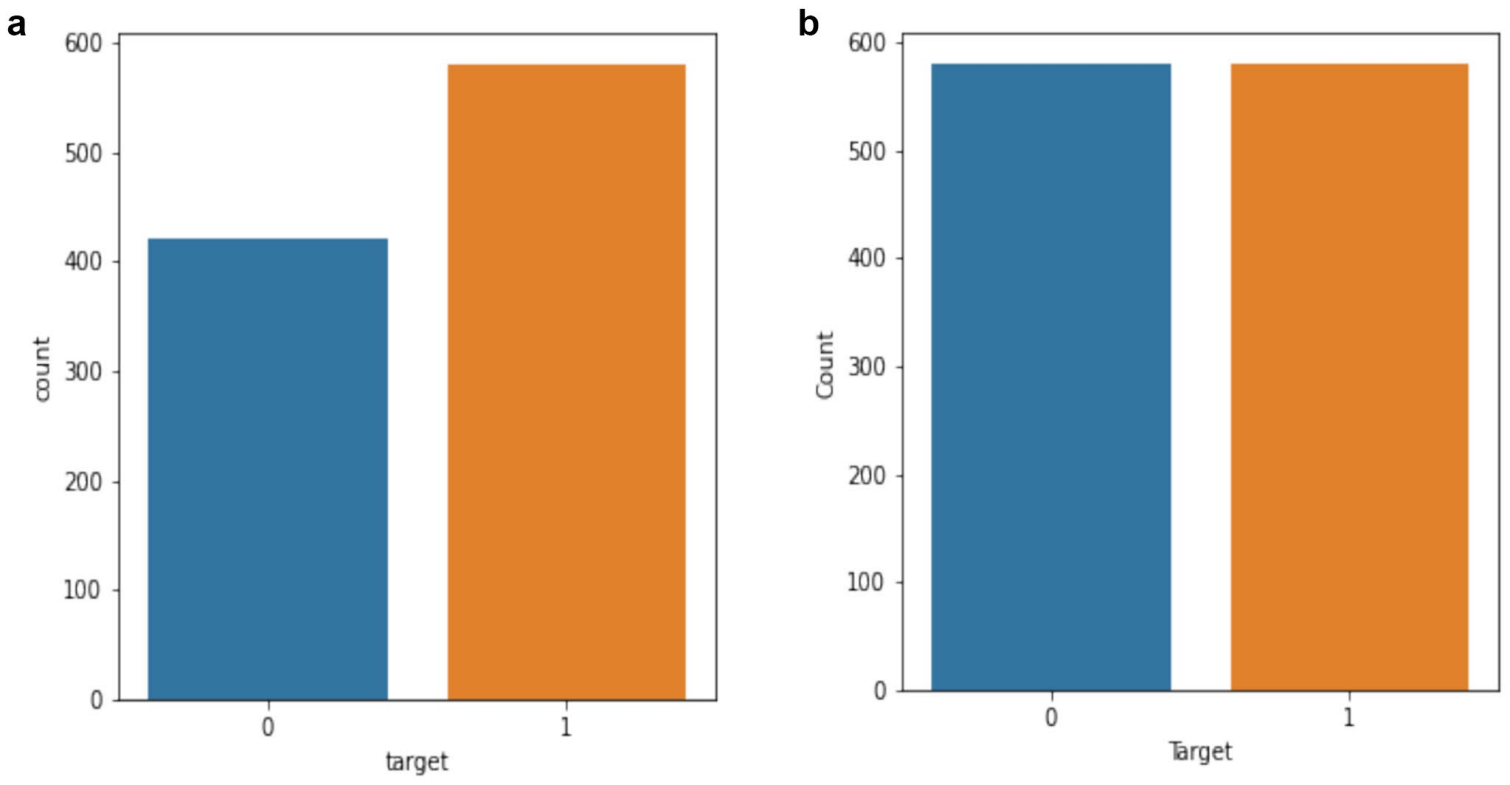
**(a)** Original dataset **(b)** augmented dataset.

While using an imbalanced data to train an ML system may improve accuracy, other metrics such as recall and precision are insufficient. Unbalanced data, if not handled properly, will produce erroneous conclusions and forecasts. As a result, these uneven features must be addressed initially in order to create a more effective system. To achieve and maintain balance across the training categories, an oversampling method known as SMOTE was used. SMOTE is an ML strategy for dealing with class imbalance that involves developing synthetic instances for the minority category. It generates new characteristics by interpolating between the existing minorities instances, allowing the model to acquire knowledge more efficiently from under-represented classes while avoiding bias towards majority classes. In order to avoid data leakage, the data was divided into training and testing sets before applying SMOTE only on the training data (see Figure 2).

### Machine learning (ML)

ML is regarded as a significant breakthrough in the evolution of AI, serving as the foundation for advanced techniques such as deep neural networks. As a subfield of AI, ML enables algorithms to learn from data and make predictions, classifications, or decisions for specific tasks without explicit programming. By identifying patterns and relationships within datasets, ML models continuously refine their performance, adapting to new and previously unseen information. This adaptive learning capability enhances accuracy and efficiency, making ML a powerful tool for solving complex problems across various domains, including intelligent disease diagnosis and predictive analytics.

#### Logistic regression (LR)

LR is a mathematical method used commonly for binary classification problems wherein the result is either 1 or 0. It uses a logistic mapping to map the link between independent variables and probability values for a certain result, producing figures that vary from 0 to 1. Logistic regression, unlike linear regression, is best suited for classified variables that are dependent, making it widely used in fields such as health, finance, and social sciences for problems such as disease detection or customer segmentation. The model is straightforward but useful, frequently acting as a basis for contrasting with higher-level models.

#### Decision tree (DT)

DT is a directed learning method used in text analysis, based on statistics, and mathematical assumptions. This approach use a categorization or predictive decision tree as a model for prediction to get understandings about a set of text data.

#### K-nearest neighbors

KNNs are types of supervised method of learning utilized in ML. KNN is an un-parametric technique, which means that it makes no assumptions about the input data’s true composition. The KNN technique attempts to categories an entirely novel input data point by comparing it with the K closest data values in the original dataset. The author specifies K, which represents the number of nearest neighbors utilized in categorization.

#### Random forest (RF)

Ensemble learning (EL) techniques, such as Random Forest (RF), are effective for classification and regression tasks. These methods function by training multiple decision trees (DTs) collectively, enhancing predictive accuracy and robustness. In classification problems, RF determines the final output based on the majority vote among individual trees. EL, as a broader strategy, integrates diverse models to tackle complex problems and optimize network performance. RF algorithms aggregate multiple decision trees, training them on different subsets of input data and averaging their predictions to improve accuracy. The approach prioritizes the most relevant predictions to enhance forecasting precision. When applied to CVD prediction using machine learning (ML), RF demonstrated superior identification performance.

#### Support vector machine (SVM)

SVM is a machine learning algorithm that uses labelled inputs to categorize new data (Ullah et al., 2024). Selection fields, or hyper planes, are used to depict decision limitations (Li et al., 2020). To separate a group of data numbers into multiple classes, a hyperspace is utilized. A Radial Basis Function (RBF) removal with a number of one was utilized. SVM attempts to categorize inputs by creating a mapping that assigns each point of a number to the appropriate class or labels, utilizing the lowest amount of bias and the highest suitable limit.

### Deep learning (DL)

Deep learning is an advanced version of ML that uses neural networks with multiple layers to handle complex data in a manner that simulates the way the human brain does work. Deep learning networks are effective tools for dealing with big training data and can acquire complex patterns by themselves, without the need for manual feature generations. On the other hand, traditional ML methods are better suited to smaller datasets since they rely on manual attribute nominations and basic computations that can often perform well with less data. While DL can improve precision as well as efficiency in real-world scenarios, it also demands a large training data and computational resources as compared to ML. An ANN is a DL approach designed to grab and recognize meaningful features and patterns from the input data and use them for future decisions. It contained interconnected nodes, or neurons, managed in an approach called layers. ANNs get knowledge from training data through training and adjust weights to improve accuracy and are widely utilized in tasks like image classification, speech processing, and NLP-based jobs. In the proposed study, a five-layer ANN with one input, three hidden, and one final layer was used, surpassing all the experimented models in this study. The ANN architecture (264–128-64) was picked through experimentation, finding a balance between model complexity and computational efficiency. Dropout and batch normalization were added to decrease overfitting and help with convergence (see Table 2).

**Table 2:** Structural info of the proposed ANN.

Layers	Neuron/value	Parameters	Value
Dense_01	264	Learning Rate (Lr)	0.001
Batch normalization		B_Size	16
Drop out	0.2	Epochs	100
Dense_01	128	E_Stop	58
Batch normalization		Activation	Relu
Drop out	0.2	Optimizer	Adam
Dense_01	64	Loss	Binary_cross_entrophy
Batch normalization		Train_Set	0.80
Drop out	0.2	Valid_Set	0.20
Dense (sigmoid)	1		

## Experiments and results

### Training environment

A training environment enables models to learn from data, using computational resources, algorithms, and tools for preprocessing, training, and evaluation. Effective research relies on diverse software and hardware. The hardware tools used in this work include a Core i7 notebook with sixteen gigabytes of RAM, a 500-gigabyte SSD, and a multi-core processing unit. To build, train, and evaluate the models, a Python tool containing several libraries such as TensorFlow, Keras, Matplotlib, Seaborn, and Scikit-learn was used, with Jupyter Notebook as the coding IDE to run Python programs.

### Performance metrics

Evaluation metrics assess a model’s reliability, efficacy, and predictive performance in research. Common metrics include accuracy, precision, recall, and F1 score, which measure how well a model generalizes to new data. Validating a model requires testing it on previously unseen data using these standardized criteria. The numerical representations of these metrics are provided below.

TP stands for true positive, FP for false positive, TN for true negative, and FN for false negative.

### Results and experiments

The goal of this research is to develop a lightweight deep learning approach for precisely and timely recognition of CVD’s initial indications in order to manage the anomaly before it causes major consequences. The study used both machine learning and deep learning approaches, which are described in detail below, along with validation findings.

#### Machine learning (ML)

**Figure 3:**
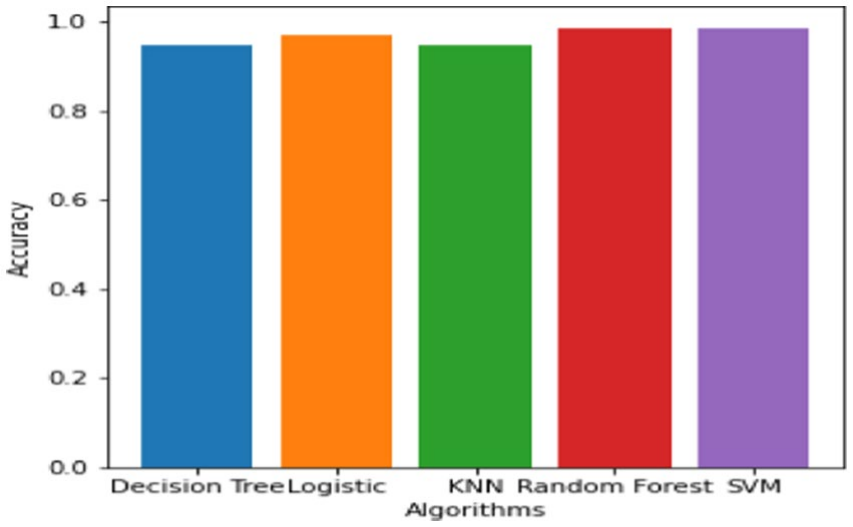
Shows comparison of ML models.

**Figure 4:**
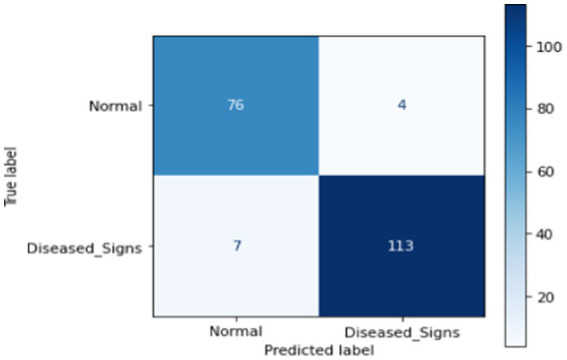
Confusion matrix of DT.

**Figure 5:**
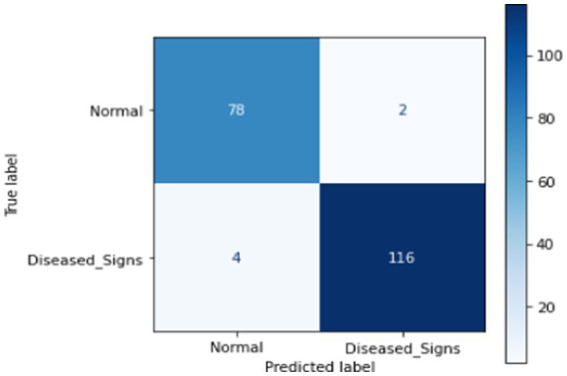
Confusion matrix of LR.

**Figure 6:**
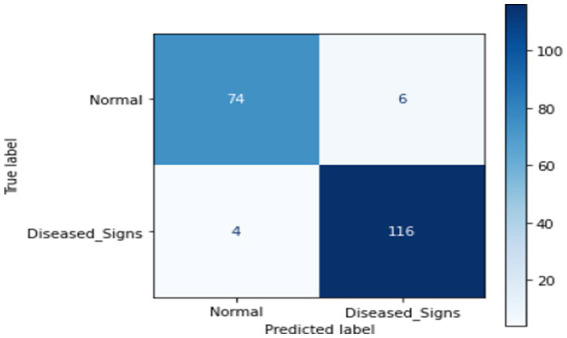
Confusion matrix of KNN.

**Figure 7:**
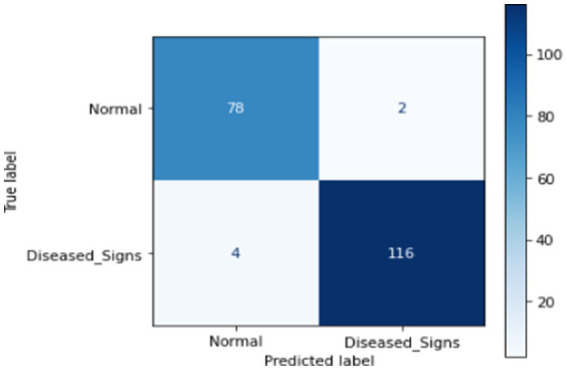
Confusion matrix of SVM.

**Figure 8:**
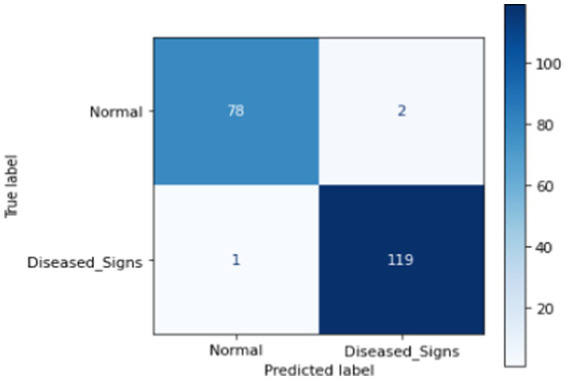
Confusion matrix of RF.

**Figure 9:**
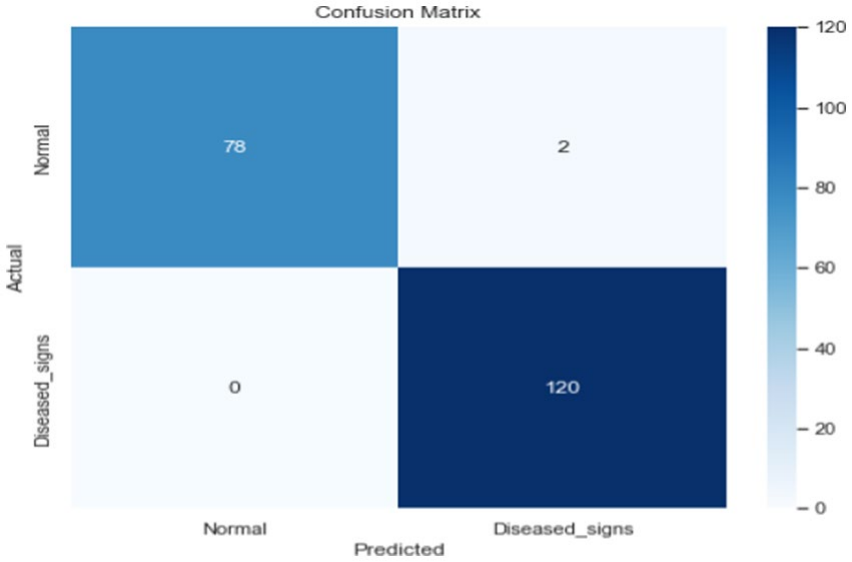
Confusion matrix of proposed DL.

This section presents ML techniques and their results using test data. Five ML algorithms, including DT, LR, KNN, RF and SVM were developed and tested on the proposed data. All of them scored well with high precision, but RF outperformed all of them with a 98.50% correctness score. Table 3 shows the detailed results of all ML models used in this study. Figure 3 shows the graphical comparison of the aforementioned models. Figures 4–9 shows the confusion matrices of the used models.

**Table 3:** Outcomes of ML approaches on test set.

S. no	Approach	Accuracy (%)	Precision (%)	Recall (%)	F_1_-score (%)
1	DT	94.50	91.57	95.00	93.25
2	LR	97.00	95.12	97.50	96.29
3	KNN	95.00	94.87	92.50	93.67
4	SVM	97.00	95.12	97.50	96.30
5	RF	98.50	98.73	97.50	98.11

#### Deep learning (DL)

The proposed deep learning (DL) approach comprises an input layer, three hidden layers with 264, 128, and 64 neurons, respectively, and an output layer. The finalized model achieved a test accuracy of 99%. Throughout this study, various DL architectures were explored, with multiple modifications to the layer structure, regularization techniques, and model width to optimize performance. The comprehensive results of the proposed DL model are presented in Table 4.

**Table 4:** Results of the proposed ANN on validation set.

S. no	Metric	Value (%)
1	Accuracy	99.00
2	Precision	100.0
3	Recall	97.50
4	F_1_-score	98.73

#### Comparison of the suggested approach with earlier SOTA techniques

In order to verify the suggested strategy’s robustness and effectiveness, it was compared to SOTA approaches in the most recent preceding literature. Table 5 compares the suggested model’s findings to earlier methodologies.

**Table 5:** Comparison of the suggested approach with earlier SOTA methods.

S. no	Ref#	Technique	Accuracy (%)
1	27	RF	96.00
2	28	RF	84.00
3	29	SVM	72.00
4	30	MLP	94.00
5	30	Neural network	92.00
6	Proposed	ANN	99.00
		RF	98.50
		SVM	97.00
		DT	94.50
		LR	97.00
		KNN	95.00

## Discussion

**Figure 10:**
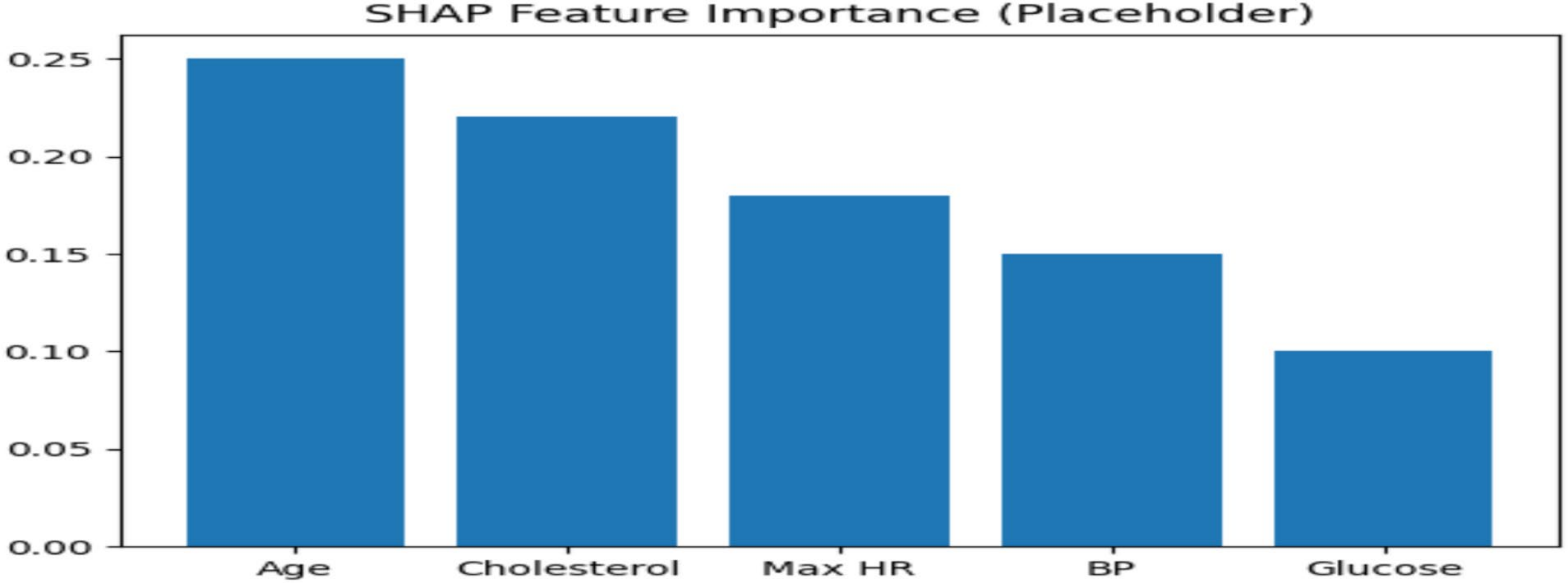
SHAP features.

**Figure 11:**
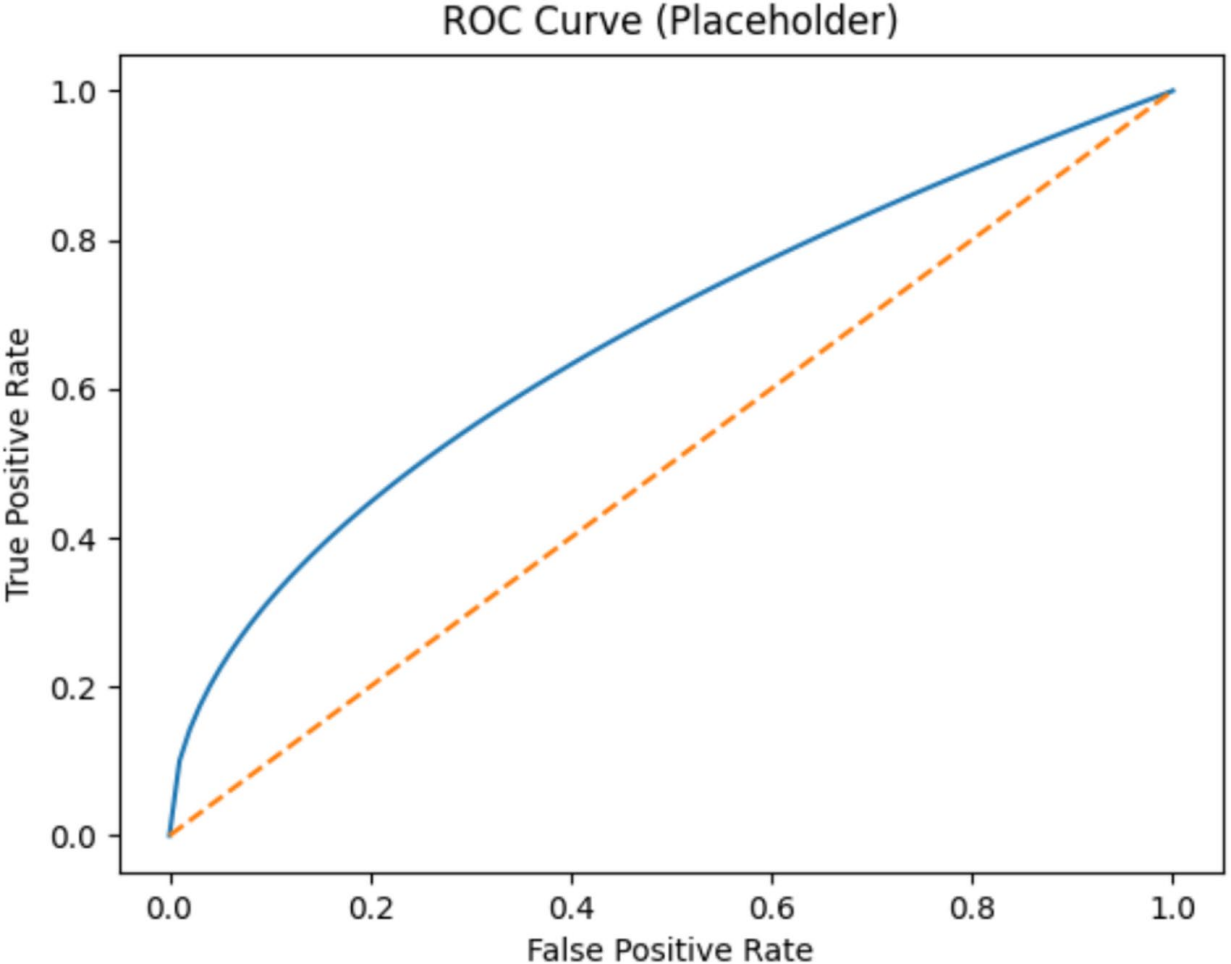
ROC curve.

Several machine learning techniques, including LR, SVM, KNN, RF, and DT, were utilized in the proposed research to detect early CVD symptoms. RF outperformed other ML algorithms with an accuracy of 98.50%. Before training, the collection of data was balanced with SMOTE to minimize bias towards the larger group, as the initial collection had 480 normal instances and 520 CVD instances. The major goal of this work was to apply and test DL models, which are recognized for producing more broad outcomes than ML models, whereas ML algorithms were utilized as a baseline method. The proposed study evaluated a number of DL architectures, including ANNs, RNNs, LSTMs, and 1D-CNNs. In comparison to ML, DL-based approaches have been described as data-hungry; hence, the training set used in this study was of a medium size and was effectively utilized when developing DL systems. We repeatedly modified the layer structure using fundamental parameters; however, it was unsuitable for the task at hand. As previously noted, these frameworks necessitate additional data due to their complicated topologies. We used a low-cost artificial neural network approach that included an input layer, three middle layers, and an output layer. Having a fairly sized data set, the suggested DL network surpassed baseline ML methods, with an accuracy of 99.00%. In addition, extending the training data is likely to improve performance. To get more reliable, robust, and generalized results, a huge number of training data must be gathered, which will be studied in the next stage of this project. The class labels were split into training (80%) and testing (20%) sets through stratified sampling before SMOTE was done. Otherwise, data leakage would have occurred. A 10-fold cross-validation was performed. The mean and the standard deviation of the results are shown. The proposed method was tested on the UCI Cleveland dataset to measure the ability of the model to generalized. Paired t-test was conducted and the improvements were statistically significant (*p* < 0.05). SHAP was utilized for explaining the model decisions and identifying which features were most important s shown in Figure 10. ROC-AUC curve indicates excellent capability of the model to distinguish between classes as shown in Figure 11. Despite the fact that the model presented achieves good prediction accuracy, the way it addresses missing data relies on standard imputation methods. Newer methods show that the absence of data may, in fact, contain clinically meaningful information. Subsequent improvements could incorporate modeling of informative missingness to increase the model’s robustness and enhance its interpretability from a clinical perspective.

## Conclusion and future work

The goal of this study is to improve prediction of CVDs by combining different ML techniques and an ANN. The research follows an organized strategy that includes data processing, model training, outcome evaluation, and comparison. The training data was leveled using the SMOTE method to make the model less biased towards the larger class. This made the values of the minority class more accurately reflect their experiences. The produced outcomes demonstrate the efficacy of the presented strategy ANN, with the highest prediction scores, indicating the utility of AI approaches in the early symptom diagnosis of CVDs. Furthermore, choosing an effective ML or DL technique is significantly influenced by the properties of the data used for training, which needs to be carefully analyzed prior to picking an approach. In future research, we intend to expand the training data with balanced classes, which will serve as a foundation for new experimentation with ML and deeper models based on DL. The deep structure of a DL model ensures greater accuracy and generalization, but it also necessitates more data instances. The project also intends to employ advanced methods based on DL and ensemble learning, as well as advanced optimization techniques, to increase the total prediction score for early indicators of CVDs.

The limited size of the dataset and the employment of SMOTE are factors that can potentially restrict the generalization capability. Larger datasets will be considered for future work. Advanced methods for handling missing data, for instance, informative missingness modeling, will be investigated in future work, where the authors will explore whether the patterns of missing values can be treated as predictive features to improve model performance.

## Data Availability

The original contributions presented in the study are included in the article/supplementary material, further inquiries can be directed to the corresponding author.
